# Magnetic activity and spectral behavior of RS CVn binary system (IM Pegasi) in the ultraviolet

**DOI:** 10.1038/s41598-025-30525-0

**Published:** 2025-12-12

**Authors:** M. R. Sanad, H. I. Abdel Rahman, W. A. Badawy

**Affiliations:** Astronomy Department, National Research Institute of Astronomy and Geophysics, Helwan, Cairo 11421 Egypt

**Keywords:** Activity, Flare, Magnetic field, Individual, IM Pegasi, Ultraviolet, Astronomy and planetary science, Climate sciences, Physics

## Abstract

We present observations from the International Ultraviolet Explorer (IUE) covering the period from 1981 to 1992 of the long-period single-lined RS Canum Venaticorum (RS CVn) binary star, IM Pegasi, to elucidate the spectral behavior and physical conditions within its atmosphere. The ultraviolet observations reveal signs of flare activity occurring in the chromosphere and transition region of the primary star. In addition to the flaring activity, the emission lines exhibit a spectrum of variations categorized as high, intermediate, and low. The relation between line fluxes and orbital phases has been established. The flaring activity was notably observed in 1985. The reddening of IM Pegasi was assessed using the 2200 Å absorption feature, yielding an estimate of E (B-V) = 0. The average mass loss rate is calculated to be approximately 1.2 × 10^− 10^ M${_ \odot }$ yr^− 1^, while the average temperature of the emitting region, determined through Planck’s equation, is approximately 9.2 × 10^4^ K. The energy associated with the flare is estimated to be around 5.6 × 10^39^ erg, and the average ultraviolet luminosity is approximately 1.23 × 10^30^ erg s^− 1^. We attribute the observed spectral variations to a cyclic behavior of the underlying magnetic field, and the flaring activity can be interpreted through the current sheet model.

## Introduction

 RS Canum Venaticorum Variable Stars (RS CVn) are binary stars of late type, exhibiting periods ranging from approximately 10 to 50 days, as inferred from the rotational variations of the spots observed in the system’s light curve. These RS CVn stars possess active chromospheres characterized by significant starspots, pronounced chromospheric plages, coronal X-ray emissions, and microwave emissions, along with heightened flares across all wavelengths, including radio, optical, ultraviolet, and X-ray bands^1–4^.

The International Ultraviolet Explorer (IUE) has been utilized to ascertain the physical conditions present in the transition region of RS CVn binary stars. Studies on rotational variation^5^ and Doppler imaging of ultraviolet emission lines^6^ suggest the existence of distinct active regions within the chromosphere and transition zones of RS CVn binary stars.

IM Pegasi is a long-period, single-lined spectroscopic binary system categorized as an RS CVn type star, located at a distance of 96.4 ± 0.7 pc. IM Pegasi comprises a K2 III primary star with an effective temperature of 4450 K and a radius of 13.3 ± 0.6 R${_ \odot }$, along with an unseen secondary companion that orbits in a circular path with an orbital period of 24.6 days^7^. Primary star exhibits significant star spots, as indicated by photometric light curves^8^ and surface maps obtained through the Doppler imaging technique^9^. The K2 III primary star displays strong chromospheric emission and rotational modulation of certain spectral lines^10–15^. The primary star of IM Pegasi is classified as a giant star with a mass of 1.8 ± 0.2 M${_ \odot }$, while the secondary companion is a dwarf star with a mass of 1.0 ± 0.1 M${_ \odot }$.

In this paper, we studied the spectral variations of the IM Pegasi binary system utilizing ultraviolet spectra acquired from the International Ultraviolet Explorer (IUE). The significant observational results from our research indicate that the fluxes of chromospheric emission lines (OI & Si III) and the fluxes of transition region emission lines (NV & CII & CIV & He II) exhibit nearly identical patterns of spectral modulations.

The structure of the paper is outlined as follows: Sect. 2 details the ultraviolet observations, Sect. 3 discusses the results and analyses related to the method for estimating reddening, as well as the spectral modulations of emission lines in the emitting regions, and Sect. 4 provides the conclusions of the paper.

## Observations and data reduction

The ultraviolet spectra with short wavelengths, acquired from the International Ultraviolet Explorer (IUE) at low resolution, have been accessed via the MAST IUE system through its main center at https://archive.stsci.edu/iue/. A comprehensive account of the ultraviolet data is provided by^16,17^.

The ultraviolet data were analyzed utilizing the standard software of IUEDAC IDL for spectral processing. The spectra were referenced to the orbital phase of the IM Pegasi binary system, employing the ephemeris provided by^18^.

1 $${\text{HJD }} =2450342.883 + 24.64880 \times {\text{ E}}$$

Table 1 presents the ultraviolet observations of IM Pegasi at low resolution. Each spectrum was examined individually within the 1150–1950 Å range to identify and eliminate noisy data, as well as data that was either overexposed or underexposed.

**Fig. 1:**
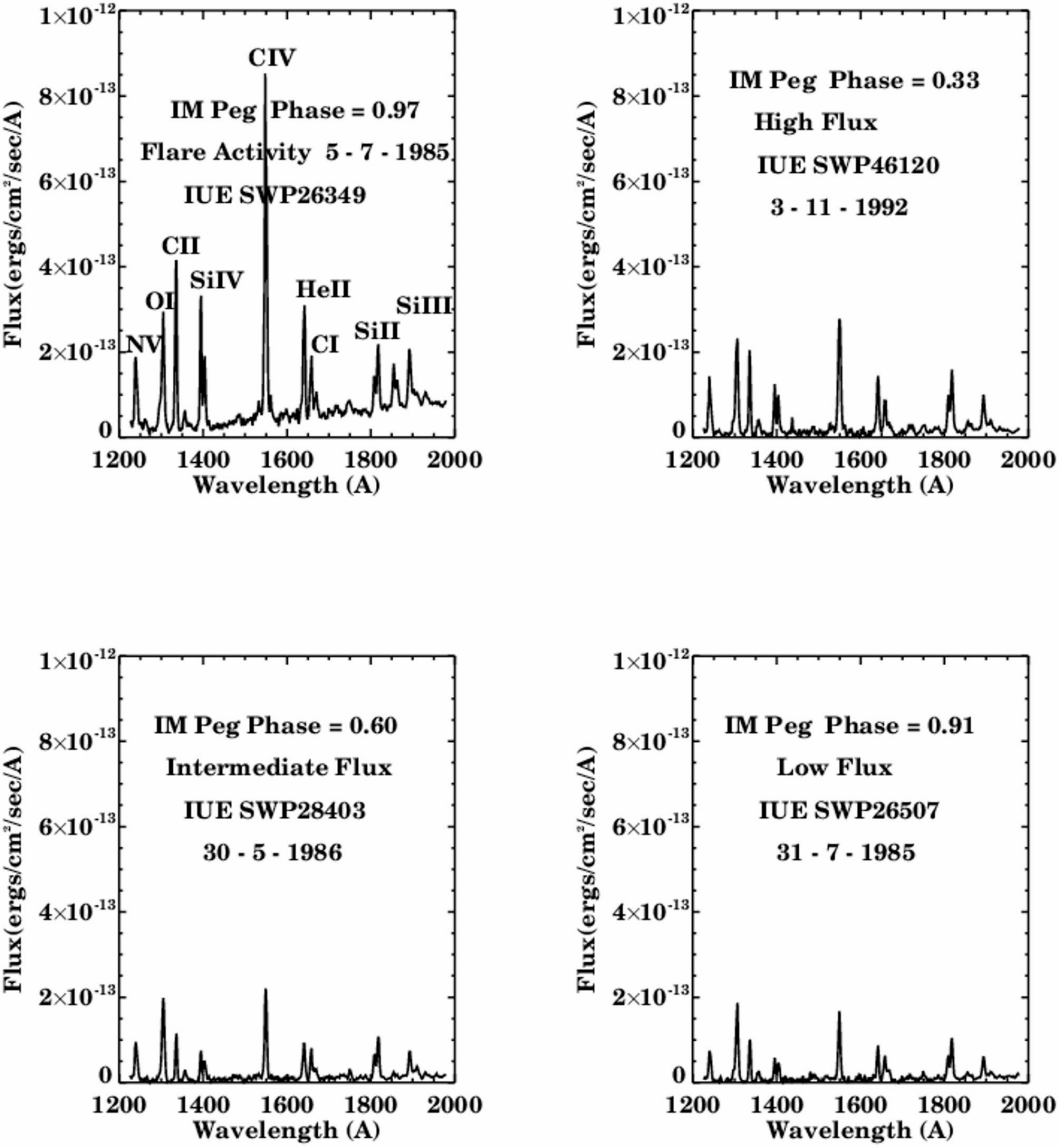
IUE spectrum of IM Pegasi with high, intermediate, and low flux at different phases and the flaring activity.

The ultraviolet observations of IM Pegasi encompass the majority of its phases. Figure 1 provides representative examples of emission lines, illustrating the variations in spectral fluxes over time and the activity associated with flares. The emission lines are derived from the chromosphere and transition region of the primary star in IM Pegasi. Ultraviolet emission lines exhibiting various ionizations, including NV at 1240 Å, Si IV at 1400 Å, and CIV at 1550 Å, have been detected in IM Pegasi, with the intensity of these emissions being evident across all phases.

**Table 1:** Journal of IUE observations of IM Pegasi.

Data ID	Dispersion	Aperture	Observation date	H.J.D.	Exposure time (s)	Phase
SWP11013 SWP26334 SWP26349 SWP26423 SWP26507 SWP26589 SWP28400 SWP28403 SWP46035 SWP46049 SWP46066 SWP46067 SWP46084 SWP46098 SWP46120 SWP46150	Low Low Low Low Low Low Low Low Low Low Low Low Low Low Low Low	Large Large Large Large Large Large Large Large Large Large Large Large Large Large Large Large	10-1-1981 2-7-1985 5-7-1985 16-7-1985 31-7-1985 11-8-1985 30-5-1986 30-5-1986 22-10-1992 24-10-1992 26-10-1992 26-10-1992 28-10-1992 30-10-1992 3-11-1992 7-11-1992	2444614.62247 2446248.96796 2446251.98479 2446262.93934 2446277.97671 2446288.90023 2446581.14623 2446581.40565 2448917.95519 2448919.94794 2448921.92591 2448922.01332 2448923.94351 2448925.92661 2448929.82282 2448933.85402	3599.844 2999.781 3899.672 2999.781 2999.781 3599.844 7199.819 3899.672 5399.627 5399.627 5399.627 1799.652 6599.755 6599.755 6599.755 6599.755	0.39 0.10 0.97 0.52 0.91 0.47 0.62 0.60 0.81 0.73 0.65 0.64 0.56 0.48 0.33 0.17

## Results and discussions

### Reddening determination of IM Pegasi

Dust grains within the interstellar medium possess a typical size that is similar to that of electromagnetic radiation. Consequently, the radiation emitted by distant stars is both scattered and absorbed by dust, resulting in the stars appearing redder than their actual color. This phenomenon is referred to as interstellar reddening and must be considered when analyzing data. The degree of reddening in the system is inversely related to the wavelength, with shorter wavelengths experiencing more reddening than longer ones. In our study, the dust reddening can be assessed through the 2200 Å absorption feature, which can then be utilized to adjust for the dust attenuation affecting the emission lines.

The method employed to ascertain the reddening of IM Pegasi relies on utilizing the most appropriate set of ultraviolet data from a Short Wavelength Prime camera (SWP) with a low resolution of (6 Å), covering the wavelength range from 1150 to 1950 Å, alongside a Long Wavelength Redundant (LWR) camera, also with a low resolution of (6 Å), spanning the wavelength range from 2000 to 3000 Å. The ultraviolet spectra for the Short Wavelength are organized into bins of 15 Å, while the Long Wavelength spectra are binned in 25 Å increments. The combination of short and long-wavelength data provides the ultraviolet spectrum profile of a reddened star, which is characterized by a distinct dip at 2200 Å.

**Fig. 2:**
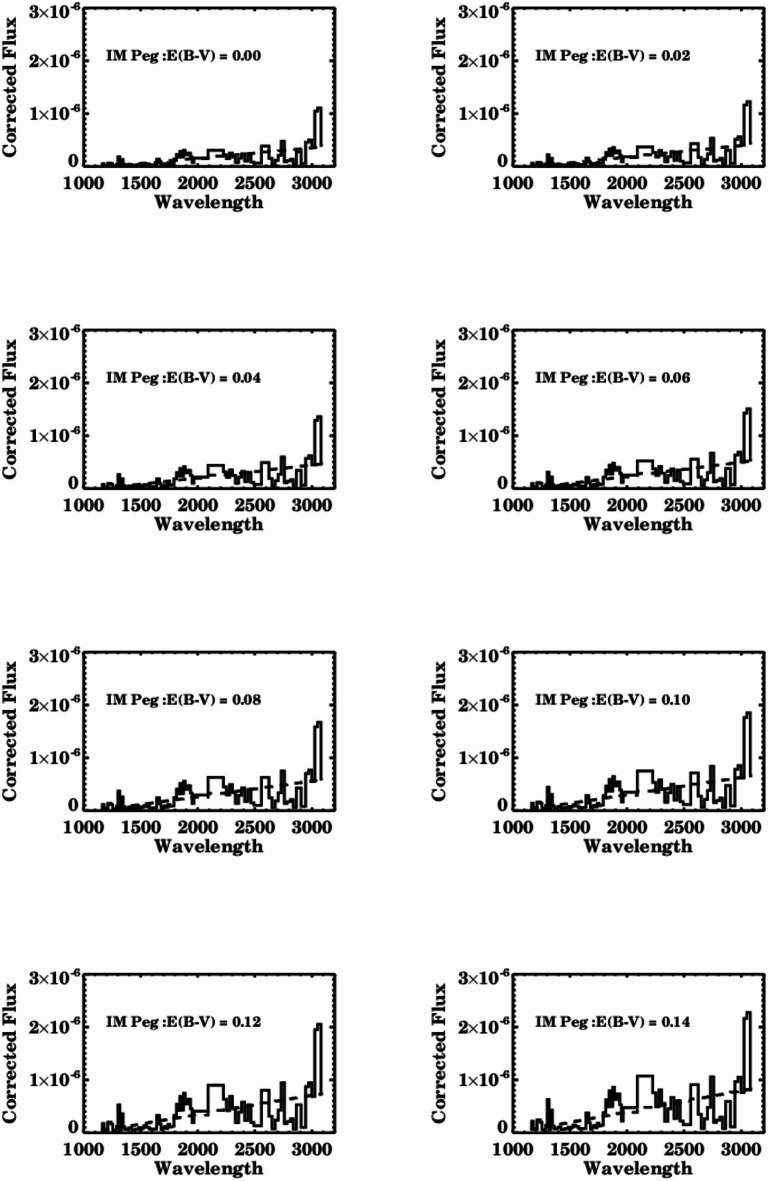
Reddening determination of IM Pegasi binary system.

The selected observations for SWP & LWP should have smooth spectrum and are in subsequent dates to be consistent in the complete range of wavelengths to give more accurate results, consequently the following subsequent ultraviolet observations are utilized in calculating the reddening value (SWP46066 – LWP24157) (SWP46084 – LWP24172) (SWP46120 – LWP24249), which leads to the most appropriate smoothing spectrum for ascertaining the reddening value. The optimal value is identified through a visual assessment of the plots to achieve the best fit for the 2200 Å absorption feature, indicating the best agreement between the observations and the standard theoretical values (represented by the dashed line). The estimated reddening value for IM Pegasi is E (B - V) = 0.00, as illustrated in Fig. 2. This determined reddening value indicates that there is no absorption or scattering occurring within the IM Pegasi binary system.

### Spectral variations of spectral lines and its source

The IM Pegasi binary system exhibits several ultraviolet emission lines that were detected using the International Ultraviolet Explorer (IUE) Short Wavelength Prime (SWP) camera. These lines are observed in the chromospheric cooler regions, including CI (1657Å), OI (1306Å), and SII (1808Å), as well as in the transition hotter areas, which feature CII (1335Å), SIV (1400Å), CIV (1550Å), He II (1640Å), NV (1240Å), and Si III] (1892 Å)^10^.

The OI, Si IV and SiII represent resonance emission lines that are excited through collisions due to the plasma’s physical conditions in the tranquil chromosphere. The He II emission line, on the other hand, is classified as a recombination line. These emission lines originate from the chromosphere and transition region of the primary star that was previously examined by^19^.

The emission lines (NV, OI, CII, CIV, He II, Si III) associated with flares, as well as those exhibiting high, intermediate, and low fluxes, demonstrate nearly identical spectral characteristics that arise from the chromosphere and transition region of the primary star. The fluxes in these emission lines were quantified by calculating the integrated area encompassed within the emission region above the continuum adjacent to the spectral line’s wings, utilizing the Gaussian profile fitting method.

**Fig. 3:**
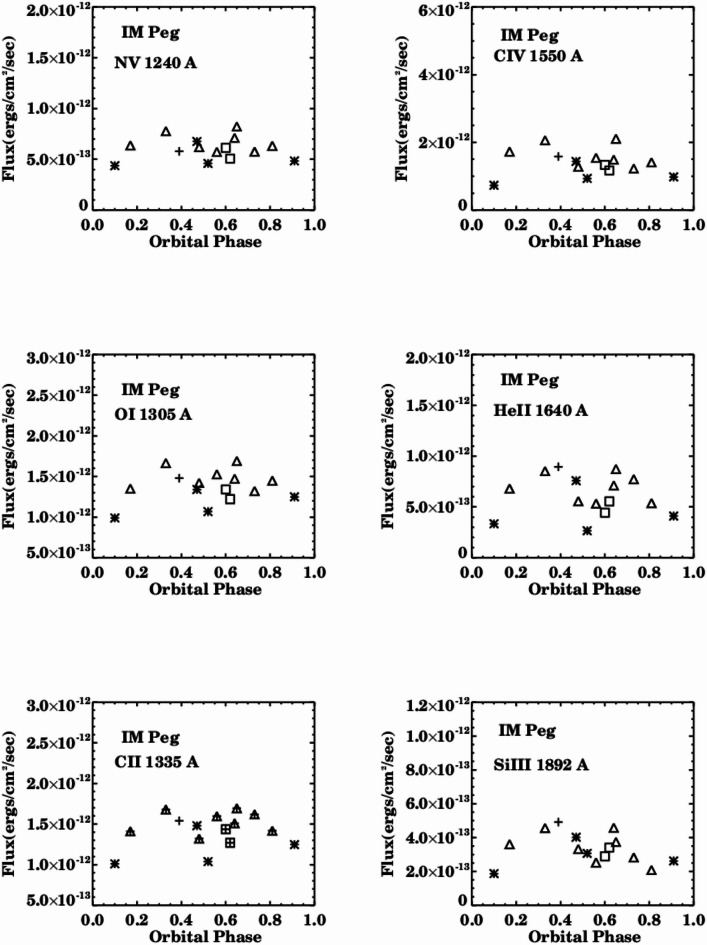
Spectral variations of the NV, OI, CII, CIV, HeII, and Si III line fluxes with phase without flaring activity. Symbol (+) represents observations in 1981, the symbol (*) represents observations in 1985, the symbol (open square) represents observations in 1986 and the symbol (open triangle) represents observations in 1992.

**Fig. 4:**
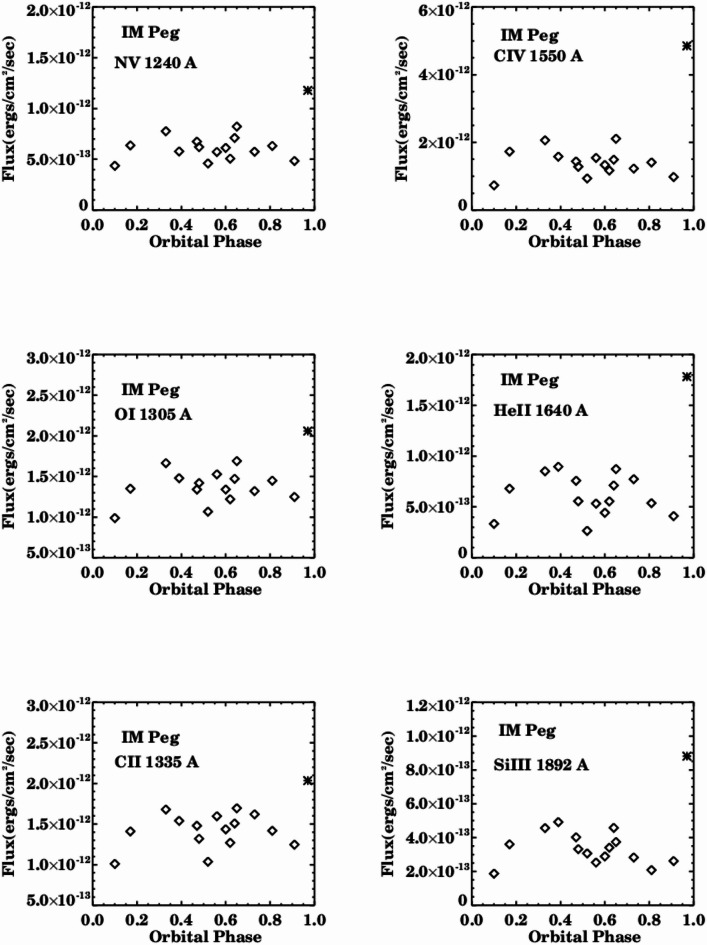
Spectral variations of the NV, OI, CII, CIV, HeII, and Si III line fluxes with phase with high value of flaring activity.

Figures 3 and 4 illustrate the fluxes of the examined emission lines in relation to orbital phases. The line fluxes exhibit correlation and variation with phase, displaying different values over short timescales of several hours and extended timescales spanning months and years during the period from 1981 to 1992. The fluxes of chromospheric lines (OI & Si III) and transition region lines (NV, CII, CIV, He II) fluctuate between high, intermediate, and low values, with significant flaring activity occurring at specific orbital phases for all emission lines.

The Tables 2, 3, 4, 5, 6 and 7 present the fluxes and values of specific emission lines for IM Pegasi. It has been observed that the spectral characteristics of IM Pegasi closely resemble those of λ Andromedae, V711 Tau, and BY Dra^20–23^. The flux of the emission lines increased to approximately three times the quiescent values, and the activity of these lines occurs around phase (0.97), which is above the typical spectral behavior, as illustrated in Fig. 4.

Huenemoerder et al.^10^conducted simultaneous IUE and optical observations over two seasons for IM Pegasi. The modulation of the ultraviolet emission lines is phase-dependent, resulting in the ultraviolet emission peaks coinciding with the minima of the visible light curve. Furthermore, the modulation of the ultraviolet emission lines intensifies with height, indicating that the emission is likely produced in loop-like structures linked to starspot regions. IM Pegasi seems to align more closely with the solar model typically employed to elucidate RS CVn activity, in contrast to more active systems.

Marsden et al.^19^identified a flare event within the short wavelength spectra of IUE. The low emission spectrum indicated enhancements reaching up to a factor of 5 in certain emission lines. The ultraviolet emission lines that are typically observable were significantly enhanced beyond the usual 30% rotational modulation, attributed to the distribution of active regions on the primary star of the IM Pegasi binary system. Both the emission fluxes from the quiescent state and the flare event were utilized to develop models depicting the variation of density and temperature with height. These models illustrate a downward shift in the transition region during the flare.

The spectral characteristics of emission lines can be interpreted in the following manner: The primary star’s atmosphere in IM Pegasi has experienced a magnetic field that is responsible for its non-radiative heating. It is proposed that magnetic fields arise from the interaction between convection and differential rotation. The rotation and convection processes in IM Pegasi lead to the concentration of magnetic fields, which in turn result in stellar activity; this interaction further enhances rotation and significantly increases magnetic activity. Magnetic fields propel hot plasma upwards, creating cooler regions that appear as dark spots in the outer layers, while also reconnecting to generate particles and magnetic free energy in the form of flares. The heating of the outer chromospheric layers can be linked to the energy released during flares. The detection of magnetic fields on the surface of the primary star has been accomplished using the Zeeman–Doppler Imaging (ZDI) technique^24^. The observed spot activity is attributed to a magnetic dynamo functioning within the convective zone, and the magnetic activity exhibits variability over time.

The fluxes of emission lines offer definitive proof of active areas within the chromosphere and transition region of IM Pegasi. These active sites are situated near the group of spots, which exhibit short-term variations in their configuration. The rise in the intensities of ultraviolet spectral lines suggests the presence of a hot region situated above a cooler spot.

The IUE observations, which show an enhancement of emission lines indicative of flaring activity, can be elucidated by the current sheet model proposed by^25,26^. In the current sheet, magnetic reconnection takes place due to instabilities that arise following the formation of the current sheet in certain regions, influenced by the plasma flow and the magnetic field configuration. These instabilities are regarded as crucial components of the initial mechanism that facilitates rapid magnetic reconnection during the compression of the magnetic field within the current sheet. As a result of this compression, magnetic shocks are generated and propagate outward, accelerating ions along their path. The plasma flow-induced compression results in temperature anisotropy, which drives the tearing instability, thereby creating magnetic regions that expand within the current sheet. This temperature anisotropy is pivotal in initiating the mechanism whereby magnetic field energy is transformed into particle kinetic energy, leading to the heating and acceleration of electrons in the compressed current sheet, accompanied by the release of magnetic energy as a stellar flare.

In summary, throughout the flare process, electrons are accelerated to high energies and travel along the flare loop, which heats the emitting transition region and produces substantial flare emissions. The ultraviolet emissions associated with flare energies can arise either from the acceleration of energetic electrons during a flare or from impact excitations caused by high-energy electrons, which can be classified as non-thermal excitation, or through thermal conduction^27^.

### Ultraviolet luminosity, flare energy, mass loss rate and temperature of the Emitting Area.

.

The ultraviolet luminosity is calculated by using the following Eq. 

2 $${L_{UV}}=4\pi F{d^2}$$

where F is the integrated flux value and d is the distance to the object 96.4 pc^15^. For the IM Pegasi binary system, utilizing the integrated fluxes of NV, OI, C II, C IV, He II, and Si III, the ultraviolet luminosities for the chosen spectral lines in various states have been calculated, as detailed in the subsequent tables.

**Table 2:** Line fluxes and ultraviolet luminosities at different states of NV.

State	Flux (erg cm^− 2^s^− 1^)	L_uv_ (erg s^− 1^)
Flare	1.18 × 10^− 12^	1.32 × 10^30^
High	8.23 × 10^− 13^	9.23 × 10^29^
Intermediate	6.75 × 10^− 13^	7.57 × 10^29^
Low	4.37 × 10^− 13^	4.90 × 10^29^

**Table 3:** Line fluxes and ultraviolet luminosities at different states of OI.

State	Flux (erg cm^− 2^s^− 1^)	L_uv_ (erg s^− 1^)
Flare	2.06 × 10^− 12^	2.31 × 10^30^
High	1.42 × 10^− 12^	1.59 × 10^30^
Intermediate	1.22 × 10^− 12^	1.36 × 10^30^
Low	9.88 × 10^− 13^	1.10 × 10^30^

**Table 4:** Line fluxes and ultraviolet luminosities at different states of CII.

State	Flux (erg cm^− 2^s^− 1^)	L_uv_ (erg s^− 1^)
Flare	2.03 × 10^− 12^	2.81 × 10^30^
High	1.41 × 10^− 12^	1.77 × 10^30^
Intermediate	1.24 × 10^− 12^	1.36 × 10^30^
Low	1.01 × 10^− 12^	6.58 × 10^30^

**Table 5:** Line fluxes and ultraviolet luminosities at different states of CIV.

State	Flux (erg cm^− 2^s^− 1^)	L_uv_ (erg s^− 1^)
Flare	4.85 × 10^− 12^	5.44 × 10^30^
High	2.11 × 10^− 12^	2.36 × 10^30^
Intermediate	1.43 × 10^− 12^	1.61 × 10^30^
Low	7.33 × 10^− 13^	8.22 × 10^29^

**Table 6:** Line flux and ultraviolet luminosities at different states of he II.

State	Flux (erg cm^− 2^s^− 1^)	L_uv_ (erg s^− 1^)
Flare	1.78 × 10^− 12^	2.00 × 10^30^
High	8.96 × 10^− 13^	1.01 × 10^30^
Intermediate	6.81 × 10^− 13^	7.63 × 10^29^
Low	4.11 × 10^− 13^	4.59 × 10^29^

**Table 7:** Values of line flux and ultraviolet luminosities at different states of Si III.

State	Flux (erg cm^− 2^s^− 1^)	L_uv_ (erg s^− 1^)
Flare	8.81 × 10^− 13^	9.88 × 10^29^
High	4.92 × 10^− 13^	5.52 × 10^29^
Intermediate	3.32 × 10^− 13^	3.72 × 10^29^
Low	2.08 × 10^− 13^	2.33 × 10^29^

Using the mass of the primary star 1.8 ± 0.2 M${_ \odot }$, and a radius of 13.3 ± 0.6 R${_ \odot }$^7^.

The rate of mass loss is calculated by using the equation^28^.

3 $$\log ({M^ \bullet })=14.02+1.24\log \left( {\frac{L}{{{L_ \odot }}}} \right)+0.16\log \left( {\frac{M}{{{M_ \odot }}}} \right)+0.81\log \left( {\frac{R}{{{R_ \odot }}}} \right) {M_ \odot } \; \text{yr}^{-1}$$

The mass loss rate is estimated to be ∼ 1.2 × 10^− 10^ M${_ \odot }$yr^− 1^.

The stored energy in the flare for the primary star is estimated by using the following equation^29^:

4 $${W_{primary}}=1.6 \times {10^{37}}\left( {\frac{l}{{{R_ \odot }}}} \right){\left( {\frac{R}{{{R_ \odot }}}} \right)^2}{\left( {\frac{{{B_{surf}}}}{{1000G}}} \right)^2} \; {\text{erg}}$$

It is important to note that only a small fraction of initially stored energy is converted to radiation, as Kopp and Poletto^30^ reported that 0.003 of the magnetic energy released during a solar flare went into heating the plasma, of course this amount will vary from flare to flare. If we assumed that a similar amount of the magnetic energy went into heating the flare plasma for the 5-7-1985 flare. By using the magnetic field strength of 50 G and l = 2R${_ \odot }$^24^ and a radius of 13.3 ± 0.6 R${_ \odot }$^7^, then we well get a total energy storage of the order of ∼ 5.6 × 10^39^ erg. This estimation shows that the binary nature of the IM Pegasi system plays a significant role in the storage of the energy of flare in the phase of pre – flare, as the existence of the second star leads to an increase of the stored energy because surface currents is generated on this star. The observable flare depends on both stored energy in the stage of pre flare and an instability, consequently the energy of the flare is the energy stored in the stage of pre flare at the moment at which an instability occurs.

The temperature of emitting area where CIV 1550 Å emission line originates is high because C IV emission line comes from carbon ions with an ionization state that requires high temperature to form and is formed in the transition region of IM Pegasi system which represents a layer of plasma between cooler chromosphere and hotter coronae. This emission line is an important indicator of the physical conditions in this dynamic emitting area where the stellar atmosphere rapidly heats up to high temperature.

The Planck’s equation can be used to calculate the energy of the transition region by using CIV emission line:

5 $$E=h\nu$$

where h is known as Planck’s constant^31,32^ and is equal to $6.626 \times 10{}^{{ - 34}}$ J. Hz^− 1^ and ν is a certain frequency in our studied ultraviolet band, at wavelength 1550 Å ($1.55 \times {10^{ - 7}}$ m) which corresponds to frequency ($1.93 \times {10^{15}}$ Hz) then E = $1.28 \times {10^{ - 18}}$ J.

By using the following equation, we can determine the temperature of the emitting area

6 $$E=kT$$

7 $$T=\frac{E}{K}$$

Where k is Boltzmann’s constant and is equal to $1.38 \times {10^{ - 23}}$ J/K, then the temperature of the transition region is.

$${\text{T}} = 9.2 \times {10^4}\;{\text{K}} \pm 500\;{\text{K}}$$

## Conclusions

The observations of the IM Pegasi binary system conducted by the International Ultraviolet Explorer indicated a similar spectral behavior among six emission lines (NV, OI, CII, CIV, He II, Si III) that originated from the chromosphere and transition region of the primary star. This emitting region is marked by fluctuations in magnetic activity, and the findings demonstrated a relation between the orbital phase and the line fluxes.

The chromosphere and transition region exhibit heightened flaring activity, with emission line fluxes increasing to approximately six times their quiescent levels, and the variations in all emission lines occur around similar phases.

The estimated physical parameters (ultraviolet luminosity, mass loss rate, stored energy during flares, and temperature) enhance the understanding of the origin of ultraviolet emission lines in the chromosphere and transition region of the primary star.

The variations of spectral behavior associated with orbital phase enhance the current sheet model, wherein magnetic reconnection takes place within the current sheet due to instabilities. These instabilities result in temperature anisotropy, which plays a crucial role in the initial mechanism that converts magnetic field energy into particle kinetic energy. Consequently, the electrons within the compressed current sheet are heated and accelerated, leading to the release of magnetic energy as a stellar flare.

## Data Availability

The used data are included in the article.
